# Supplementary figures and images for: Clinical connectivity map for drug repurposing: using laboratory results to bridge drugs and diseases
Source: BMC Med Inform Decis Mak. 2021 Sep 24;21(Suppl 8):263. doi: 10.1186/s12911-021-01617-4 (PMC8461864; doi:10.1186/s12911-021-01617-4)

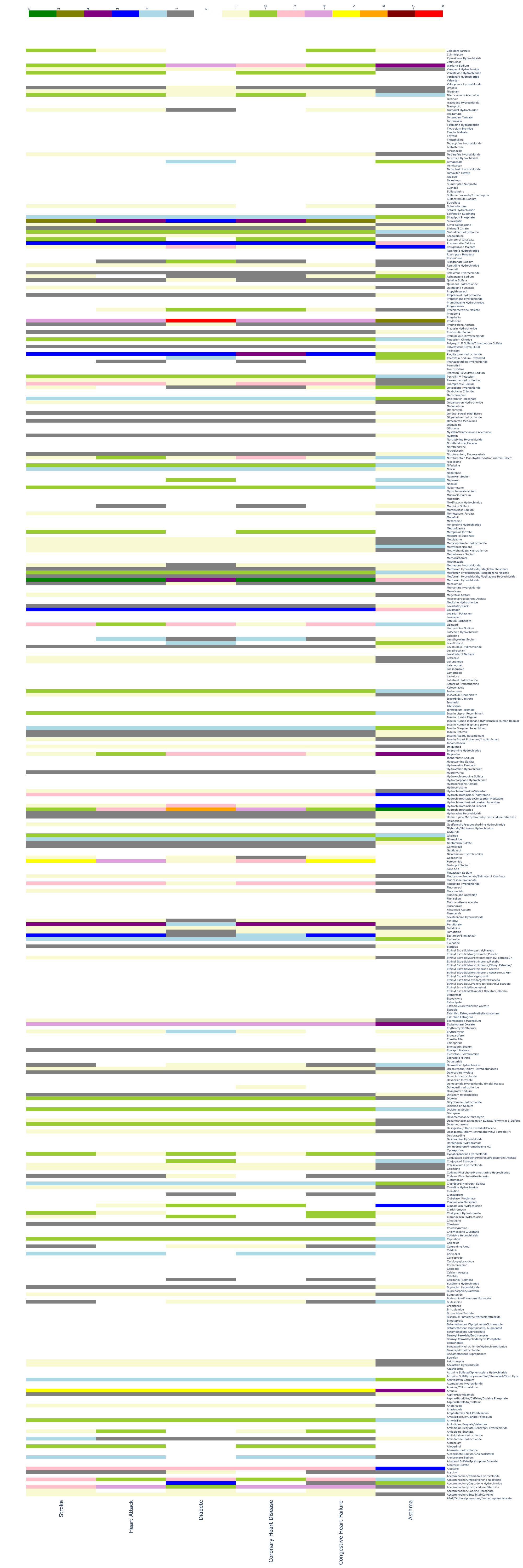

Supplement: Supplementary file 3 — Additional file 3: Figure S3 Detailed Drug-Disease Heat Map. We transform the repurposing possibility score table into heat map and present it in this figure. This version includes the repurposing possibility scores of all the drug-disease pair. [file 12911_2021_1617_MOESM3_ESM.png]
