# Supplementary material for: Clinical connectivity map for drug repurposing: using laboratory results to bridge drugs and diseases
Source: BMC Med Inform Decis Mak. 2021 Sep 24;21(Suppl 8):263. doi: 10.1186/s12911-021-01617-4 (PMC8461864; doi:10.1186/s12911-021-01617-4)
Supplement: Supplementary file 5 — Additional file 5: Table S1. Laboratory Result List. This table includes the name of 35 kinds of laboratory result involved in ourexperiments and their corresponding NHANES code. Figure S1. Disease Clinical Variable Statistics. The figure present number of diseases will increase (Up) or decrease(Down) the level of each clinical variables. X-axis is the name of eachclinical variable, Y-axis is the number diseases. Blue bar stands for the “Up”relation, red bar stands for the “Down” relation. Figure S2. Drug Clinical Variable Statistics. The number of drugs will increase (Up) or decrease (Down) the level ofeach clinical variables. X-axis is the name of each clinical variable, Y-axis isthe number diseases. Blue bar stands for the “Up” relation, red bar standsfor the “Down” relation. [file 12911_2021_1617_MOESM5_ESM.pdf]

Laboratory Test	NHANES CODE
Albumin (Serum)	LBXSAL
Alkaline Phosphatase	LBXSAPSI
Alanine Aminotransferase	LBXSATSI
Aspartate Aminotransferase	LBXSASSI
Basophils	LBDBANO
Basophils/100 Leukocytes	LBXBAPCT
Bilirubin	LBXSTB
Calcium	LBXSCA
Chloride	LBXSCLSI
Cholesterol	LBXTC
Eosinophils	LBDEONO
Eosinophils/100 Leukocytes	LBXEOPCT
Erythrocyte Distribution Width	LBXRDW
Erythrocytes	LBXRBCSI
Globulin	LBXSGB
Glucose Serum	LBXGLU
Glycohemoglobin	LBXGH
HDL	LBDHDD
Hematocrit	LBXHGB
Hemoglobin	LBXHCT
LDL	LBDLDL
Leukocytes	LBXWBCSI
Lymphocytes	LBDLYMNO
Lymphocytes/100 Leukocytes	LBXLYPCT
Monocytes	LBDMONO
Monocytes/100 Leukocytes	LBXMOPCT
Neutrophils	LBDNENO
Neutrophils/100 Leukocytes	LBXNEPCT
Platelets	LBXPLTSI
Potassium	LBXSKSI
Protein	LBXSTP
Sodium	LBXSNASI
Triglycerides	LBXTR
TSH	LBXTSH1
Urea Nitrogen	LBXSBU

Table S1. Laboratory Test List. This table includes the name of 35 kinds of laboratory test involved in our experiments and their corresponding NHANES code.

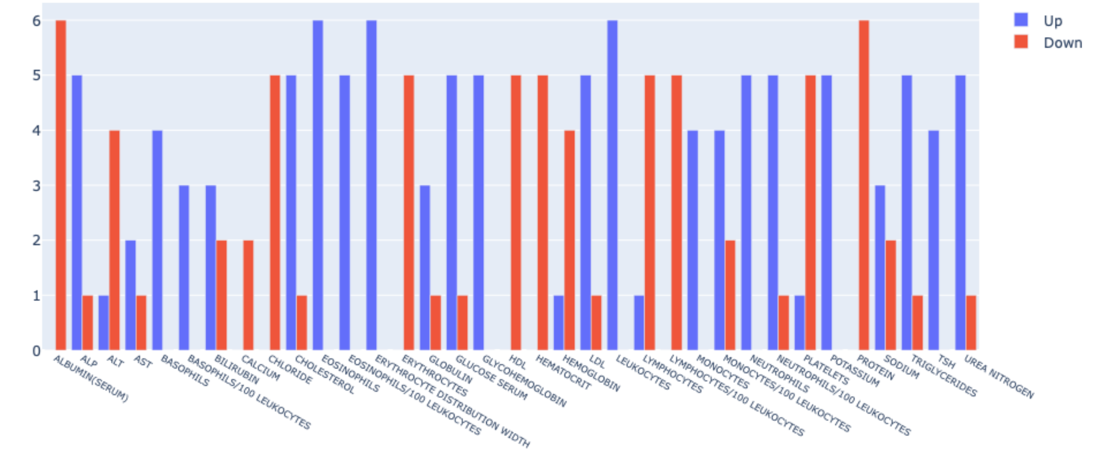

Figure S1. Disease-Clinical Variable Statistics. The figure present number of diseases will increase (Up) or decrease (Down) the level of each clinical variables. X-axis is the name of each clinical variable, Y-axis is the number diseases. Blue bar stands for the "Up" relation, red bar stands for the "Down" relation.

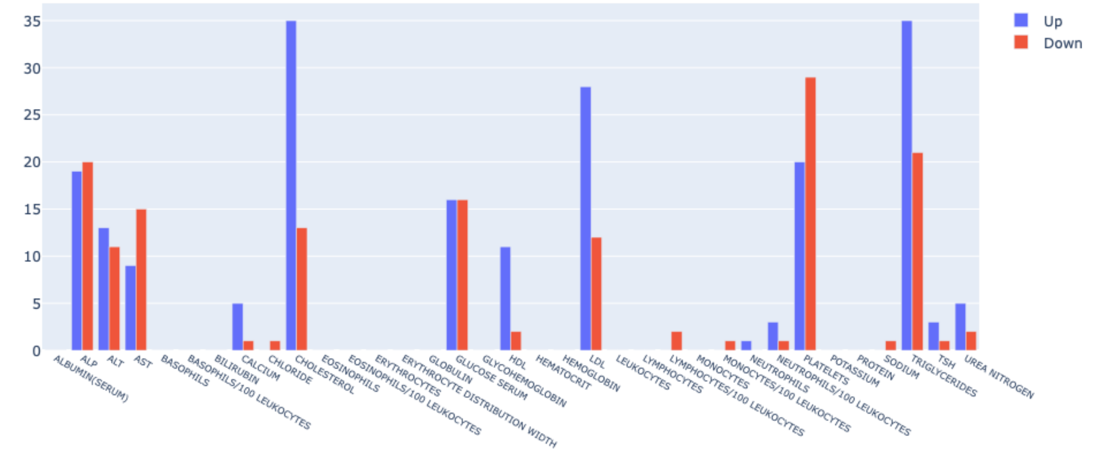

Figure S2. Drug-Clinical Variable Statistics. The number of drugs will increase (Up) or decrease (Down) the level of each clinical variables. X-axis is the name of each clinical variable, Y-axis is the number diseases. Blue bar stands for the "Up" relation, red bar stands for the "Down" relation
